# Cocrystals of β-Sitosterol with Propionic Acid Improve Postprandial Lipid Response and Long-Term Adaptation to Obesogenic Diets in Hamsters, Surpassing the Effects of Commercial β-Sitosterol

**DOI:** 10.3390/nu18132146

**Published:** 2026-07-02

**Authors:** Mariona Palou, Bàrbara Reynés, Rafel Prohens, Fernando Barrera, Andreu Palou-March, Andreu Palou, Francisca Serra

**Affiliations:** 1 Group of Nutrigenomics, Biomarkers and Risk Evaluation (NuBE), University of the Balearic Islands, 07122 Palma, Spain; mariona.palou@uib.cat (M.P.); a.palou-march@uib.es (A.P.-M.); andreu.palou@uib.es (A.P.); francisca.serra@uib.es (F.S.); 2 Institut d’Investigació Sanitària Illes Balears (IdISBa), 07010 Palma, Spain; 3 CIBER de Fisiopatología de la Obesidad y Nutrición (CIBEROBN), 28029 Madrid, Spain; 4 Artificial Intelligence Research Institute of the Balearic Islands (IAIB), 07122 Palma, Spain; 5 Alimentómica S.L. (Spin off no. 001 from UIB), 07120 Palma, Spain; 6 Laboratory of Organic Chemistry, Faculty of Pharmacy and Food Sciences, University of Barcelona, Avda. Joan XXIII, 08028 Barcelona, Spain; rafel_prohens@ub.edu; 7 Center for Intelligent Research in Crystal Engineering, S.L. (CIRCE), 07121 Palma, Spain; fbarrera@circescientific.com

**Keywords:** sterols, crystal engineering, dyslipidemia, obesogenic-diet protection, nutraceuticals, cardiovascular disease

## Abstract

**Objectives:** This study investigated the short- and long-term metabolic effects of two β-sitosterol with propionic acid cocrystals (CCA and CCB) compared with commercial β-sitosterol in male hamsters. **Methods**: Five experiments were conducted, including three acute and two chronic dietary interventions. Acute experiments evaluated the metabolic response to a single dose of commercial β-sitosterol (S) or its cocrystals (513 mg/kg) administered with a pork-fat load following 40% caloric restriction. Blood samples were collected before and 5 h after the lipid challenge. Long-term studies assessed daily supplementation with CCA (264 mg/kg) or CCB (at two doses, 114 and 342 mg/kg) during exposure to high-fat (HF) or Western diets (WD), respectively, for 21 days. **Results**: Acutely, CCA attenuated postprandial triglyceride increases in adult animals, while CCB induced a reduced rise in postprandial cholesterol across the three studies compared to S. Young animals treated with CCA and CCB exhibited a reduction in postprandial glucose levels. Long-term, in HF-fed hamsters, CCA lowered plasma cholesterol and triglyceride levels on day 14 and, at the endpoint, decreased liver weight and increased circulating sitosterol levels, unlike its commercial counterpart. In WD-fed animals, CCB supplementation dose-dependently elevated plasma sitosterol levels. At the low dose, CCB treatment reduced body weight gain and cholesterol increase and improved adipocyte morphology, whereas the high dose reduced diet-induced hepatic detrimental outcomes, showing a stronger hepatoprotective effect compared to S-treated animals. Together, these results indicate improved postprandial lipid handling in the short-term and a modulation of metabolic adaptation to obesogenic diets in the long-term. **Conclusions**: β-sitosterol with propionic acid cocrystals improved metabolic responses more effectively than commercial β-sitosterol, enhancing bioavailability and potential therapeutic value against diet-induced metabolic disturbances.

## 1. Introduction

According to the World Health Organization, cardiovascular diseases (CVD) account for approximately 18 million deaths per year globally, representing nearly one-third of all deaths worldwide [1]. Dyslipidemia, particularly elevated total cholesterol and LDL levels, low HDL levels, and postprandial dyslipidemia, plays a central role in the development and progression of CVD [2,3].

Phytosterols, including plant sterols and stanols, are naturally occurring steroidal compounds structurally similar to cholesterol, differing mainly in their aliphatic side chains and the presence or absence of specific double bonds (e.g., β-sitosterol vs. sitostanol) [4]. They are abundant in plant foods, particularly in vegetable oils, nuts, and avocados, but cannot be synthesized by humans. Thus, their presence in circulation depends solely on dietary intake [4]. Clinical trials and meta-analyses have consistently demonstrated that daily consumption of phytosterols (from 0.5 to 9 g a day) significantly reduces circulating total and LDL-cholesterol levels [5,6,7]. These effects have been recognized by regulatory agencies, including the FDA and EFSA, which support their use in food and food supplements for LDL-cholesterol reduction [8,9].

Their primary hypocholesterolemic mechanism is the competitive displacement of cholesterol from mixed micelles in the intestinal lumen, reducing its absorption. Additional mechanisms may involve modulation of intestinal transporters and hepatic cholesterol metabolism [10]. Nevertheless, phytosterols have extremely low aqueous solubility and negligible oral bioavailability, limiting their potency and broader metabolic benefits [11,12]. Improving these physicochemical properties remains a key challenge.

Crystal engineering has emerged as a promising strategy to improve the physicochemical properties of poorly soluble compounds, for instance, carbamazepine–nicotinamide or itraconazole–succinic acid. Cocrystals, formed through the supramolecular assembly of an active ingredient with a coformer, can modify solubility, dissolution kinetics, and stability without altering the chemical identity of the active ingredient [13,14]. However, cocrystal formation remains inherently difficult to predict and is not always achievable under experimental conditions. Moreover, anticipating the physicochemical properties of a given cocrystal before its experimental preparation continues to be a significant challenge. Despite these limitations, the development of computational approaches has opened new avenues in this field. In particular, the pioneering work by Hunter and co-workers on virtual cocrystal screening has demonstrated that intermolecular interaction propensity can be used to rationally identify suitable coformers for cocrystal formation [15]. This methodology, further applied and expanded in subsequent studies [16], has successfully enabled the discovery of new cocrystals, reducing experimental trial-and-error and accelerating the screening process [17]. Despite these advances, identifying optimal coformers and experimental crystallization conditions still often requires considerable time, effort, and resources. Nevertheless, successful examples have demonstrated substantial improvements in pharmacokinetic performance and in vivo efficacy across various classes of drugs and nutraceuticals [18,19], such as cocrystals of carbamazepine, itraconazole, curcumin, and resveratrol.

Although the potential of cocrystallization in pharmaceutical applications is well established, its application to nutraceuticals such as phytosterols remains relatively unexplored. Previous studies demonstrated that β-sitosterol can form well-defined multicomponent crystal structures through supramolecular interactions, providing detailed structural insights into these systems. However, these studies focused primarily on crystal packing and intermolecular interactions, and did not address whether such modifications translate into improved biological or metabolic outcomes in vivo [20,21]. Propionic acid was selected as one of the coformers based on its physicochemical properties. It is a small, biocompatible molecule able to establish strong hydrogen bonds with the hydroxyl group of β-sitosterol, facilitating stable crystal packing. In addition, it is naturally present in foods [22], making it particularly suitable for nutraceutical development. These works provided the first structural basis supporting the use of crystal engineering strategies to modify and potentially improve the properties of phytosterols, but the in vivo metabolic effects of these cocrystals have not been evaluated yet. Interestingly, the study by Tang et al. (2025) [23] is, to our knowledge, the only study to date investigating the in vivo effects of phytosterol cocrystal supplementation. They reported that bile acid–based phytosterol cocrystals enhanced aqueous solubility, dissolution rate, and oral bioavailability, as well as potentiated lipid-lowering effects in high-fat (HF) fed rats [23].

These findings highlight growing interest in applying cocrystallization to nutraceuticals whose clinical potential is constrained by poor solubility. For β-sitosterol, enhancing dissolution and intestinal accessibility is expected to strengthen its interference with cholesterol absorption and potentially extend its metabolic impact. However, evidence regarding the in vivo metabolic efficacy of phytosterol cocrystals remains scarce, and the extent to which crystal engineering can systematically improve lipid profiles or other cardiometabolic parameters has yet to be established.

In this context, the novelty of the present study lies in providing a comprehensive in vivo evaluation of two distinct β-sitosterol–propionic acid cocrystals, structurally characterized, linking crystal engineering with metabolic functionality. By combining three acute studies and two long-term interventions in a hamster model of diet-induced dyslipidemia, we hypothesize that β-sitosterol cocrystals would exhibit superior lipid-lowering efficacy and broader metabolic benefits compared with natural commercial β-sitosterol.

## 2. Materials and Methods

### 2.1. Preparation of β-Sitosterol–Propionic Acid Cocrystals

CCA and CCB cocrystals were prepared following procedures described elsewhere [20]. On the one hand, the propionic acid cocrystal hydrate (β-sitosterol: propionic acid: H_2_O; 2:1:1, CCA) was obtained by antisolvent precipitation using water from a propionic acid solution of β-sitosterol at 25 °C. On the other hand, the propionic acid cocrystal hydrate (β-sitosterol: propionic acid: H_2_O; 4:1:1, CCB) was obtained via solvent-mediated transformation of β-sitosterol in a propionic acid–acetone suspension at 25 °C for 1 day. The crystal structures and physicochemical characterization of both cocrystals, including single-crystal X-ray diffraction and thermal analysis, have been previously reported in detail [20,21].

### 2.2. Animal Design

Male golden Syrian hamsters from Charles River Laboratories (Barcelona, Spain) and Janvier Labs (Le Genest-Saint-Isle, France) were purchased for the five different experiments (Exp) designed. The animal protocol was in accordance with the guidelines set by the Bioethical Committee of the University of the Balearic Islands regarding the use and care of laboratory animals and was approved by this institution (Resolution Number 46/10/15, November 2015). All the animals were individually housed in cages at 22 °C with a 12:12 h light/dark cycle. Before the treatment period, all the animals had free access to a standard chow diet (Panlab, Barcelona, Spain). The composition of the diets used in the five experiments is shown in Supplementary Table S1. The total number of animals was 96.

#### 2.2.1. Short-Term Experiments

Three acute studies (Exp 1.1, 1.2, and 2) were performed in young (6 weeks old, once) and adult hamsters (16 weeks old, twice, testing two lots), maintained under a standard chow diet (Table 1).

Each experimental setup included: a control group (C) receiving the vehicle; a reference group (S) receiving commercially available β-sitosterol (Sigma-Aldrich, Madrid, Spain, ref. 85451); a group receiving the cocrystalline form of β-sitosterol with propionic acid A (cocrystal A, CCA); and a group receiving the cocrystalline form of β-sitosterol with propionic acid B (cocrystal B, CCB). The cocrystals and the commercial reference of β-sitosterol were tested by providing an equivalent amount of β-sitosterol. The dose tested in the three acute studies was the human equivalent dose corresponding to 4.16 g β-sitosterol/day (513 mg/kg hamster), which corresponds to 2.5 times the human dose of 1.66 g a day. In Exp 1.1 and 1.2, we used the same lot of compounds and tested the influence of age (Table 1). In Exp 2, we used adult animals and studied the effect of a second lot of cocrystals.

Animals were subjected to a 40% calorie restriction (CR40) for 24 h before treatments, and, after this period, blood samples (100 µL) were collected from the saphenous vein, using heparinized glass capillaries. Then, both cocrystals and commercial β-sitosterol were administered by placing the material inside the animal cage, mixed with a pork fat oral load (2.5 g/kg). Control animals received the same amount of pork fat. Animals were first acclimatized to the procedure by being offered the pork fat portion for three consecutive days to be familiarized with its flavor and texture. After this acclimation, a wash-out period of at least 15 days was implemented before the experiments. On the testing day, animals freely consumed the small food portion provided.

Five hours after the lipid load (T5), blood collection depended on the experiment: in Exp 1.1, animals (n = 7/group) were euthanized by sodium pentobarbital (80 mg/kg), and blood was collected by cardiac puncture from the left ventricle using heparin in NaCl (0.9%) as an anticoagulant for further analyses; in Exp 1.2 (n = 6/group) and Exp 2 (n = 7–8/group), blood at T5 was collected from the saphenous vein, using heparinized glass capillaries, and animals were left alive to be integrated into Exp 3. Heparinized glass capillaries and heart blood samples were centrifuged at 1000× *g* to collect plasma and stored at −20 °C for analytical determinations.

Before the acute assays, an oral fat tolerance test (OFTT) was performed on a small set of another cohort of animals to determine the best time (the peak) to perform the acute challenge. For this, 4 animals were CR40 for 24 h (from 8:00 am to 8:00 am), and then a load of 2.5 g/kg body weight of lard pork was orally given to the hamsters as described above. Blood samples were taken from the saphenous vein into heparinized containers before fat load (at time zero, corresponding to 24 h after CR40) and at 2, 3, 5, 7, and 9 h thereafter. Plasma TG levels were measured. The challenge test performed 5 h after the oral fat load constitutes a good method to check the lipidemia response in the short term [3].

#### 2.2.2. Long-Term Experiments

Two chronic studies (Exp 3 and Exp 4) were designed to characterize the long-term effect of cocrystal treatments in animals exposed to hypercaloric diets (Table 1). As in acute experiments, a control group (C), β-sitosterol groups (S), and the groups with the tested compound (either CCA or CCB) were processed in parallel within each study.

In Exp 3, the CCA (n = 8) or commercial β-sitosterol (n = 8) was given daily, mixed with a rodent HF diet powder providing 5.2 kcal/g and containing 60% kcal from fat (Ref D12492, Research Diets Inc., New Brunswick, NJ, USA) for 21 days. The dose tested was the human equivalent dose corresponding to 2.3 g β-sitosterol/day (264 mg/kg hamster). Handmade pellets were deposited in the cage of the animals, allowing ad libitum intake. The average estimation of food intake was calculated daily, and adjustments in the amount of diet supplied were performed to get the planned dose of the treatment. Control animals (n = 8) were also studied, which received the pellets without treatment. Before (D1) and after 13 days of treatment (D14), blood was collected from the saphenous vein, processed, and stored as in acute experiments for analytical determinations. At day 22 (D22), animals were sacrificed, and blood was obtained from cardiac puncture.

Exp 4 was performed in animals receiving a Western diet (WD) providing 4.7 kcal/g and 40% and 43% kcal from fat and carbohydrates, respectively (Ref D12079B, Research Diets Inc., New Brunswick, NJ, USA). WD was supplied ad libitum during the 14 days before initiating the cocrystal treatment to induce obesity. In addition, during this period, animals were adapted to be offered three times a week with a dose of the planned vehicle (0.1 g of easy-to-spread butter) to get used to its taste and the route of administration (directly in the mouth by using a syringe without a needle, by voluntary intake). Supplementary Table S2 shows the basal body weight as well as glucose, cholesterol, and triglyceride plasma levels of the animals before the beginning of the WD in Exp 4. After 14 days under WD feeding, animals were separated into 5 groups according to their subsequent daily oral treatment for 21 days: C, control animals, treated with the vehicle (n = 12); S1 and S3, animals treated with commercial β-sitosterol at two doses (n = 8 per dose group); and CCB1 and CCB3, hamsters supplemented with the cocrystal B at the equivalent two doses of the commercial reference (n = 8 per dose group). The two doses tested were the human equivalent doses corresponding to 1 g β-sitosterol/day (114 mg/kg hamster, S1) or 3 g β-sitosterol/day (342 mg/kg hamster, S3). Before (D1), after 12 days of treatment (D13), and 21 days of treatment (D22), blood was collected from the saphenous vein, in heparinized glass capillaries, processed, and stored as in acute experiments for analytical determinations.

In both long-term experiments (Exp 3 and 4), at D22, the animals were sacrificed by using lethal doses (50–80 mg/kg body weight) of pentobarbital (Dolethal, Farma Vet Balear SL., Palma, Spain) in ad libitum conditions during the first two hours of the beginning of the light. Arterial blood was collected by cardiac puncture from the left ventricle. Afterward, retroperitoneal white adipose tissue, liver, heart, muscle, and kidney were rapidly removed and weighed, frozen in liquid nitrogen, and stored at −80 °C. Blood samples were collected using EDTA (Exp 3) or heparin in NaCl (0.9%) (Exp 4) as anticoagulant and were centrifuged at 1000 *g* for 10 min at 4 °C to collect plasma.

### 2.3. Measurement of Circulating β-Sitosterol in Hamsters

Circulating β-sitosterol in plasma was determined by Gas chromatographic techniques combined with a flame ionization detector (GC-FID) (CG-7890A, Agilent Technologies, Barcelona, Spain) as previously described [24]. Briefly, 100 μL of plasma mixed with a constant amount of α-cholestane as an internal standard was saponified with ethanolic KOH. The unsaponifiable fraction was extracted three times with hexane, dried under nitrogen, derivatized to trimethylsilyl ethers, and analyzed using an Agilent 7890A gas chromatograph (Agilent Technologies) equipped with an HP-5MS capillary column and coupled to FID. Quantification was performed using calibration curves generated from derivatized β-sitosterol standards and the β-sitosterol/α-cholestane peak area ratio.

### 2.4. Measurement of Circulating Parameters (Alkaline Phosphatase, Alanine and Aspartate Transaminases, Cholesterol, Glucose, and Triacylglycerols) in Hamsters

Blood glucose concentration was measured using an Accu-Chek Glucometer (Roche Diagnostics SL, Barcelona, Spain). Circulating cholesterol, tissue transaminases, alkaline phosphatase, HDL, and triacylglycerol (TG) were measured using commercial enzymatic colorimetric kits (BioSystems, Barcelona, Spain; Química Clínica aplicada S.A., Tarragona, Spain; Sigma-Aldrich Química SA, Madrid, Spain, respectively). The levels of LDL were calculated using Friedewald’s Formula [25].

### 2.5. Quantification of Liver Lipid Content

Total hepatic lipids from Exp 4 were extracted from 300 mg of liver tissue using the Folch et al. method [26]. In brief, the tissue was homogenized in chloroform-methanol (2:1, *v*/*v*), filtered, and phase-separated with 0.9% NaCl. The organic phase was collected and evaporated, and lipids were quantified.

### 2.6. Histological Analysis

Morphological characterization of the steatosis degree in the liver and adipocyte morphology in the retroperitoneal depot (rWAT) from Exp 4 animals was studied. For this purpose, tissue samples were fixed by immersion in 4% paraformaldehyde in 0.1 M sodium phosphate buffer, pH 7.4, overnight at 4 °C, dehydrated in a graded series of ethanol, cleared in xylene, and embedded in paraffin blocks for light microscopy. Five-micrometer-thick sections of tissues were cut with a microtome, mounted on slides, and stained with hematoxylin/eosin. For each sample, 2–3 photos were digitized from light microscopy (Zeiss Axioskop 2 microscope) connected with AxioCam ICc3 digital camera (Carl Zeiss, S.A., Barcelona, Spain). In liver sections, the presence of fatty vesicles was studied by using the software AxioVision 40V 4.6.3.0 (Carl Zeiss, Imaging Solutions GmbH, Germany). For rWAT, the diameter of all adipocytes from each photo of rWAT was measured by using the software AxioVision 40V 4.6.3.0. Distributions of adipocyte size were obtained from individual data on cell sizes. Image analysis from all samples and groups was examined blindly.

### 2.7. Statistical Analysis

Data are presented as mean ± standard error of the mean (SEM).

Normality and homogeneity of variance were evaluated through the Shapiro–Wilk and Levene tests, respectively. When necessary, data were logarithmically transformed before analysis.

When the same parameter has been measured at different time-points within the same study, a repeated measures ANOVA has been performed, taking into account the factors of time (T) and treatment group (G). Then, the differences between more than three groups at the same time-point were analyzed using one-way ANOVA followed by least significant difference (LSD) post-hoc comparison. Subsequent planned pairwise comparisons to evaluate differences between two groups were conducted using Student’s *t*-test or Paired *t*-test. Cell diameter distributions of rWAT adipocytes were compared between experimental groups using chi-square tests. Mean frequencies per diameter class were calculated across animals within each group, and diameter distributions were subsequently grouped into quartiles before analysis. The analyses were performed with SPSS for Windows (IBM SPSS Statistics 28.0.0.0, Chicago, IL, USA). The threshold of significance was defined at *p* < 0.05 and is indicated when different.

## 3. Results

### 3.1. Short-Term Experiments

Body weight after 24 h of CR40 (T0) and circulating levels of glucose, cholesterol, and triglycerides (TG) at T0 and after 5 h of the fat load (T5) of the Exp 1.1, 1.2, and 2 are shown in Table 2.

**Figure 1:**
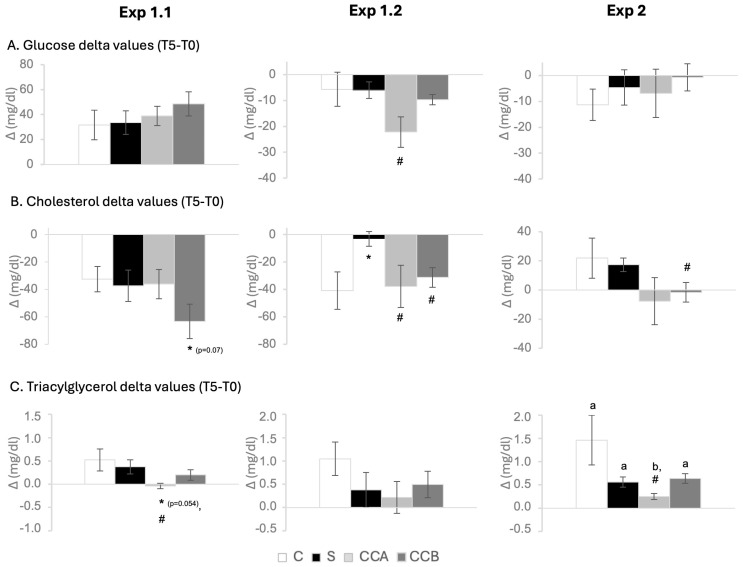
Changes in blood parameters between 24 h of 40% calorie restriction (T0) and after 5 h of the fat load (T5) in hamsters of the Exp 1.1, 1.2, and 2. Delta (Δ) values were calculated as the difference between the two values for each animal. Data are means ± s.e.m. (n = 6–8). Statistics: one-way ANOVA followed by LSD *post hoc* test was performed (data not sharing a common letter are significantly different). Single comparisons between different groups were performed by Student *t*-test: *, different from controls; #, different from S group. Abbreviations: control (C), sitosterol (S), cocrystal A (CCA), and cocrystal B (CCB).

Delta (Δ) values, calculated as the difference between the two values for each animal (T5 minus T0), for glucose, cholesterol, and TG of the Exp 1.1, 1.2, and 2 are illustrated in Figure 1.

Body weights did not differ between groups in any of the three acute experiments.

Blood glucose levels increased over time in Exp 1.1 and decreased in Exp 1.2 and 2 (repeated measures ANOVA). Interestingly, the decrease in glucose levels in young animals (Exp 1.2) after treatment (T5 vs. T0) was significant by Paired Student’s *t*-test only in animals treated with CCA and CCB, but not in the control or S groups (Table 2). Furthermore, within the same experiment, CCA caused a greater decrease in blood glucose after treatment (Figure 1A) compared to the S group (Student’s *t*-test).

Regarding cholesterol levels, they decreased over time in Exp 1.1 and 1.2, regardless of the treatment (T5 vs. T0, repeated measures ANOVA). Strikingly, in Exp 2, the S treatment resulted in a significant increase in plasma cholesterol levels by paired Student’s *t*-test (T5 vs. T0), but not in the other treated groups (Table 2).

Notably, CCB induced a consistently smaller increase in cholesterolemia (ΔCHOL, T5–T0) across the three acute experiments (Figure 1B). In adult animals, CCB produced a reduced rise in cholesterol levels compared with controls in Exp 1.1 (*p* = 0.070, Student’s *t*-test) and compared with the S group in both Exp 1.2 and Exp 2 (Student’s *t*-test). In young animals (Exp 1.2), groups of control and CCA also exhibited smaller cholesterol delta values than the S group (Student’s *t*-test) (Figure 1B).

Interestingly, animals treated with CCA showed lower circulating TG at T5 compared to controls, S-treated, and CCB-treated in Exp 2 (one-way ANOVA). In young animals (Exp 1.2), of note, by paired Student’s *t*-test, plasma TG levels were significantly higher at T5 compared to the levels at T0 only in the control group, but not in the rest of the animals treated with sterols (Table 2). Moreover, treatment with CCA induced, in adult animals, a clear resistance to the fat-load–induced rise in triglycerides, as reflected by a markedly lower TG delta value (T5–T0) (Figure 1C). Animals treated with CCA showed a reduced ΔTG compared with both C and S groups (*p* = 0.054 and *p* < 0.05 vs. C and S, respectively, in Exp 1.1; *p* < 0.05 vs. both groups in Exp 2; one-way ANOVA) (Figure 1C). In Exp 2, the ΔTG in the CCA group was also significantly lower than that observed in animals receiving CCB (one-way ANOVA) (Table 2).

### 3.2. Long-Term Experiments

#### 3.2.1. Results of Exp 3

**Figure 2:**
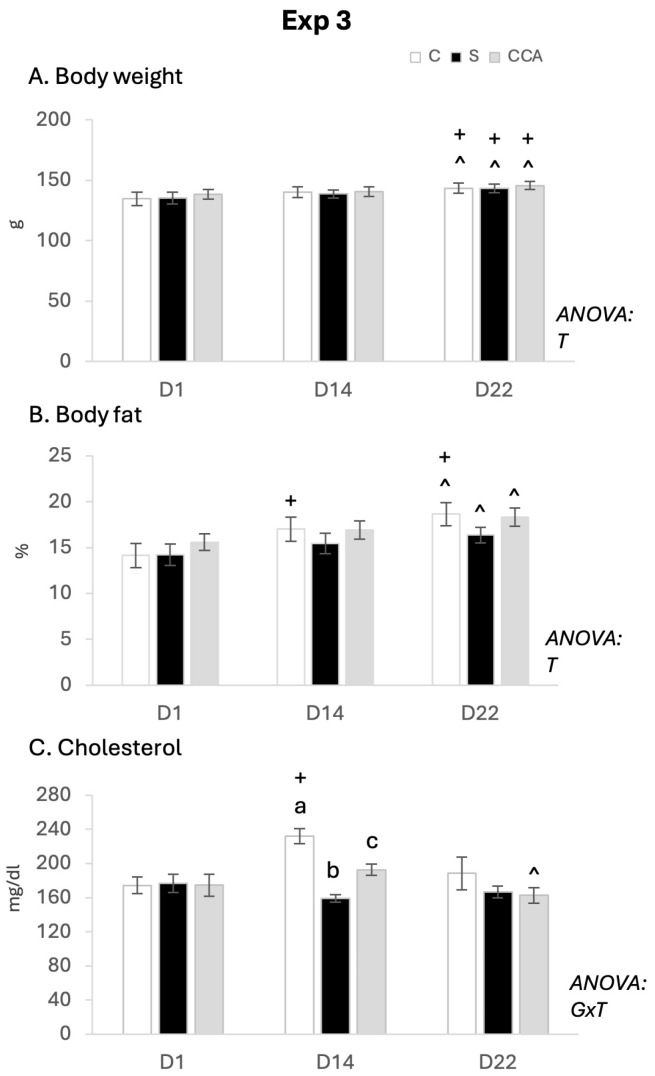
Body weight, body fat content, and plasma cholesterol levels at day (D)1, D14, and D22 in hamsters from Experiment 3. Data are means ± s.e.m. (n = 8). Statistics: repeated measures ANOVA was performed to analyze the effects of treatment group (G) and/or the three days studied (T). Within the same day, one-way ANOVA followed by LSD *post hoc* test was performed (data not sharing a common letter are significantly different); Single comparisons between different groups were performed by Student *t* test: Single comparisons between different days of measurement within the same group were performed by Paired *t* test: +, different from D1; ^, different from D14. Abbreviations: control (C), sitosterol (S), and cocrystal A (CCA).

Figure 2 shows the evolution of body weight, percentage of body fat content, and circulating cholesterol levels of the 3 groups of animals from Exp 3 (controls, S, and CCA).

No significant differences were found in body weight or body fat between the three groups of animals on any of the days studied. Body weight (Figure 2A) increased significantly with exposure time to the HF diet in all three animal groups (repeated measures ANOVA and paired *t*-test D22 vs. D1, D13 vs. D1). However, it was noteworthy that, although the evolution of body fat content (Figure 2B) followed the same pattern of increase over time in response to HF diet in all three animal groups (repeated measures ANOVA), body fat on day 22 compared to day 1 was higher and only significant by paired Student’s *t*-test in the controls, but not in those hamsters treated with S or CCA. Interestingly, cholesterol levels (Figure 2C) were significantly lower on day 14 in hamsters treated with S or CCA compared to their controls (one-way ANOVA), with the decrease being greater in the animals treated with S. No significant differences were found on day 1 or day 22 among the treatments. Notably, the evolution of cholesterol over time was different among the 3 groups of animals (interactive effect between treatment time and group of treatment, repeated measures ANOVA). It is remarkable that the increase on day 14 compared to day 1 in cholesterol levels was greater and significant by Paired *t*-test only in the control animals, but not in those treated with CCA or S. Moreover, the levels of cholesterol decreased at day 22 compared to day 14 only in the hamsters treated with CCA (Paired *t*-test). Curiously, cholesterol levels on day 22 were not different from those on day 1 by Paired *T*-test in any of the 3 groups of animals, not even in the controls (Figure 2C). Supplementary Figure S1 shows the delta value of cholesterol plasma levels and % of body weight and body fat gain for Exp 3.

**Figure 3:**
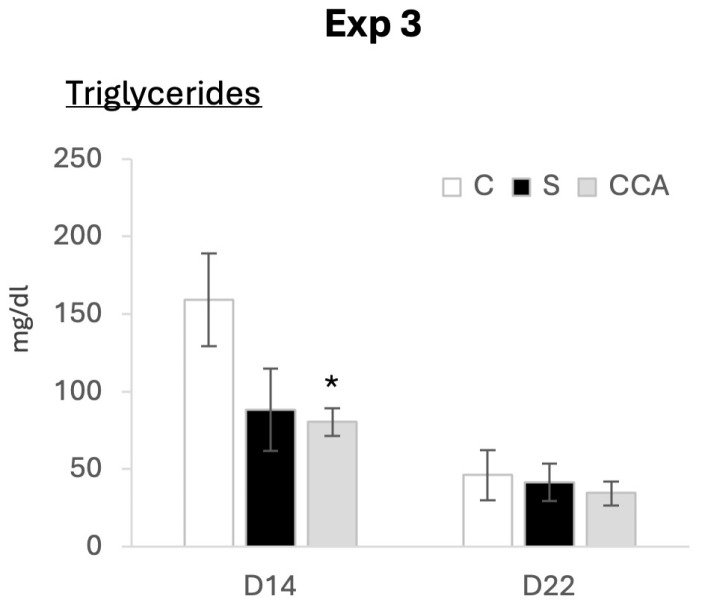
Plasma triglyceride levels at day (D)14 and D22 in hamsters from Experiment 3. Data are means ± s.e.m. (n = 8). Statistics: Single comparisons between different groups were performed by Student’s *t*-test: *, different from controls. Abbreviations: control (C), sitosterol (S), and cocrystal A (CCA).

TG levels on day 14 (peripheral blood) and at the end of the study (cardiac blood) are shown in Figure 3. Notably, on day 14, TG levels were significantly lower in animals treated with CCA, but not in those treated with S, compared to controls (Student’s *t* test). There were no significant differences on day 22.

Table 3 shows blood glucose and tissue weights obtained at the endpoint (D22) of Exp 3. Interestingly, animals treated with CCA, but not those treated with S, had a lower liver weight (relative to BW) compared to the controls (Student’s *t*-test). There were no significant differences in glucose levels or in the weights of the rWAT, kidney, and heart among the different treatments.

**Figure 4:**
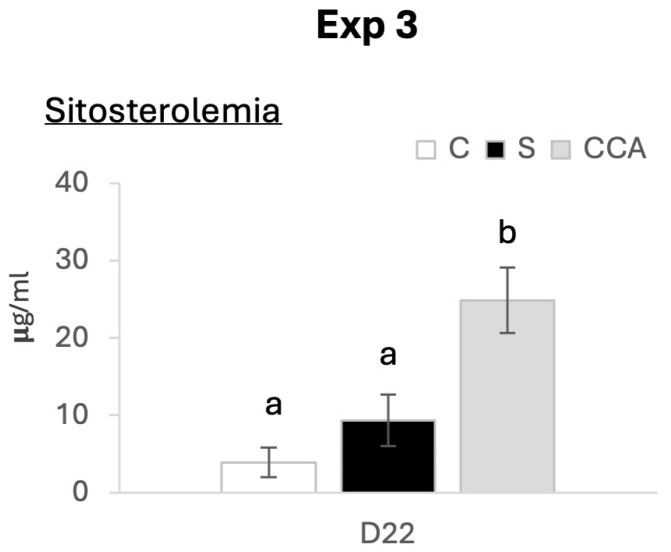
Plasma Sitosterol levels at the end of the study (day 22) in hamsters from Experiment 3. Data are means ± s.e.m. (n = 8). Statistics: one-way ANOVA followed by LSD *post hoc* test was performed (data not sharing a common letter are significantly different). Abbreviations: control (C), sitosterol (S), and cocrystal A (CCA).

Sitosterol levels on day 22 are shown in Figure 4. Notably, circulating sitosterol levels were significantly higher in animals treated with CCA compared to the controls and those treated with S (one-way ANOVA). In contrast, circulating sitosterol levels were not different between control hamsters and those treated with S. These findings are consistent with an enhanced oral absorption of sitosterol with CCA; however, the magnitude of the increase remains within the expected physiological range.

#### 3.2.2. Results of Exp 4

Body weight, body fat content, and the levels of glucose, TG, and cholesterol at day 1 (14 days after WD and before initiating treatments), day 13, and day 22, as well as the plasma levels of HDL and LDL at the endpoint from Exp 4, are shown in Table 4.

No significant differences were found in body weight and body fat among the five groups of animals, nor in glucose, triglyceride, and HDL levels, on any of the days studied. Cholesterol levels on day 13 were significantly lower in the 4 groups of animals treated with phytosterols compared to their controls (one-way ANOVA), differences that disappeared at the end of treatment. The animals treated with CCB1 showed a tendency to lower LDL levels compared to the controls (*p* = 0.064, Student *t* test). As expected, body weight increased over time regardless of treatment (repeated measures ANOVA). However, it should be noted that in animals treated with S3, CCB1, or CCB3, the increase did not reach statistical significance until day 22, and was not evident at day 14, in contrast to the control group and S1-treated animals (Student *t* test). Although overall weight gain from day 1 to day 22 was not prevented by the treatments, the delayed onset of a significant increase may suggest a transient effect of these treatments on body weight progression.

**Figure 5:**
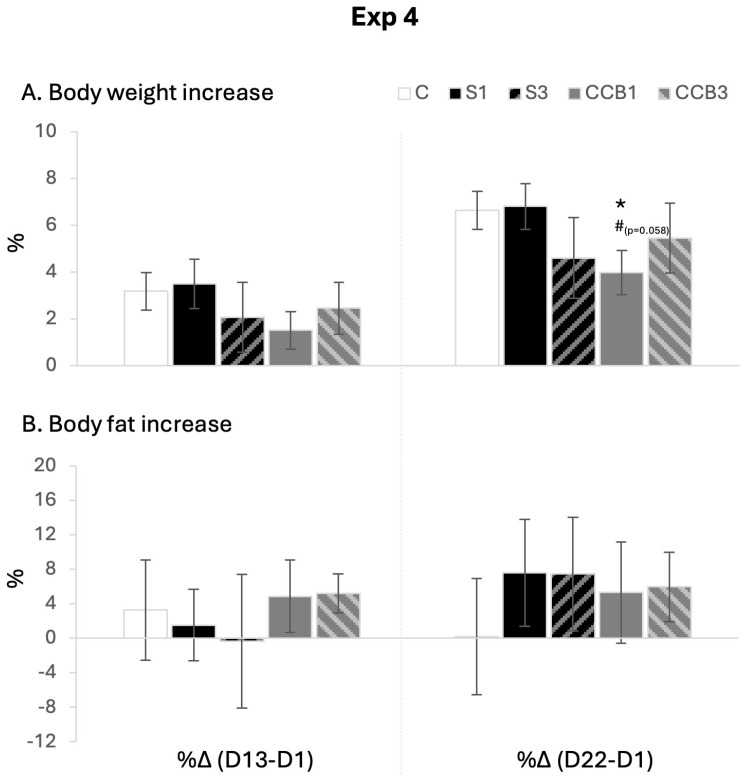
Percentage of body weight and body fat increase from the midpoint (day 13) or endpoint (day 22) to the day of beginning the treatments (day 1) in hamsters from Experiment 4. Data are means ± s.e.m. (n = 8–12). Statistics: Single comparisons between different groups were performed by Student *t* test: *, different from controls; #, different from S group. Abbreviations: control (C), sitosterol (S) at both doses (1 and 3), and cocrystal B (CCB) at both doses (1 and 3).

The percentage increase in body weight and body fat between day 13 or day 22 and day 1 is shown in Figure 5. Interestingly, the animals treated with CCB1 showed a lower percentage increase in body weight compared to the controls (Student *t* test) and the animals treated with S1 (*p* = 0.058, Student *t* test).

**Figure 6:**
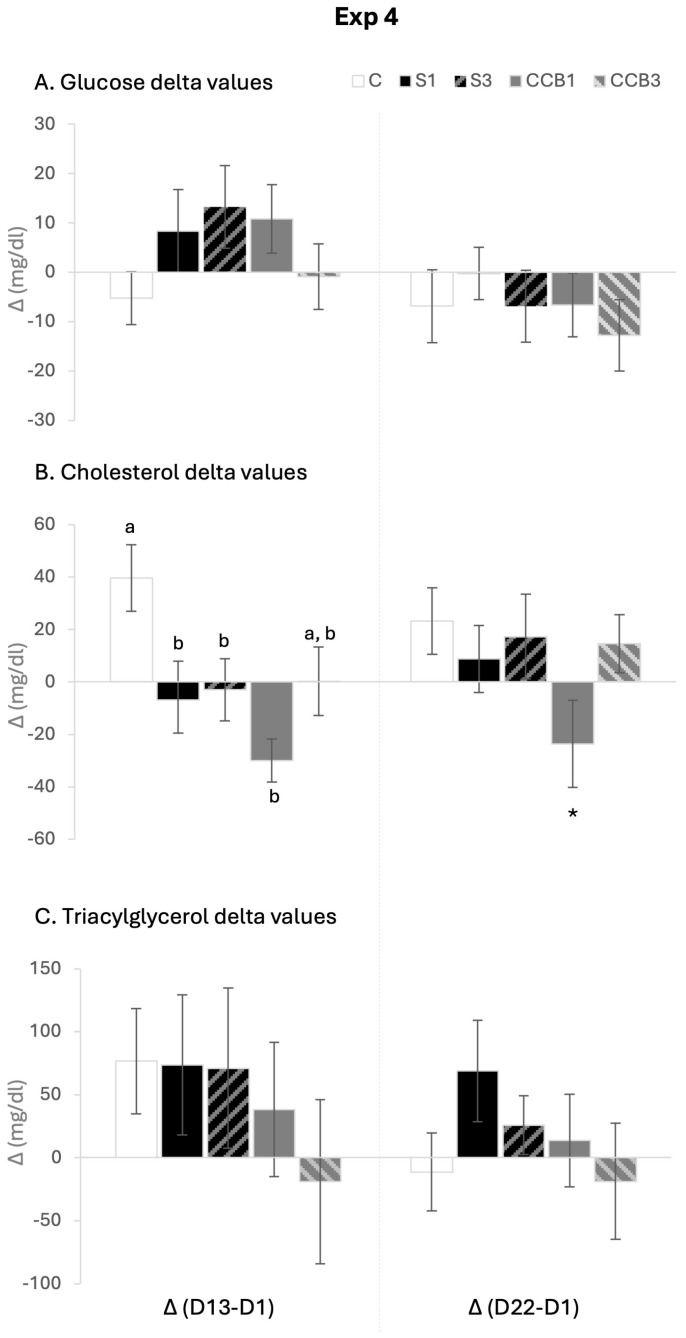
Changes in blood parameters between the midpoint (day 13) or endpoint (day 22) and the day of beginning the treatments (day 1) in hamsters from Experiment 4. Delta (Δ) values were calculated as the difference between the two values for each animal. Data are means ± s.e.m. (n = 8–12). Statistics: one-way ANOVA followed by LSD *post hoc* test was performed to compare all the groups (data not sharing a common letter are significantly different); Single comparisons between different groups were performed by Student *t* test: *, different from controls. Abbreviations: control (C), sitosterol (S) at both doses (1 and 3), and cocrystal B (CCB) at both doses (1 and 3).

Figure 6 shows the delta values for circulating glucose, cholesterol, and triglyceride levels on days 13 and 22 compared to day 1 in the animals of Exp 4. Animals treated with S3 showed at day 13 a trend toward higher delta values for circulating glucose compared to controls (*p* = 0.066, Student’s *t*-test). Moreover, treatment with sitosterol either commercial or cocrystals resulted in decreased cholesterol delta values on day 13 compared to controls, although this reduction was only partial in the CCB3 group (one-way ANOVA). On day 22, only animals treated with CCB1 maintained significantly lower cholesterol delta values compared to controls (Student’s *t*-test). No significant differences were found for triglycerides.

Supplementary Table S3 shows the weights of the tissues collected at sacrifice (d22) relative to the body weight of the animals in Exp 4. No significant differences were found between the different animal groups. 

**Figure 7:**
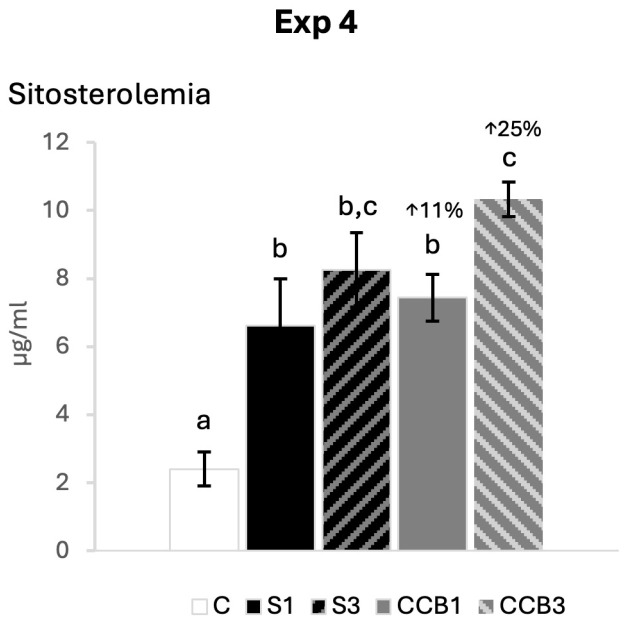
Plasma Sitosterol levels at the end of the study (day 22) in hamsters from Experiment 4. Data are means ± s.e.m. (n = 8–12). Statistics: one-way ANOVA followed by LSD *post hoc* test was performed (data not sharing a common letter are significantly different). Arrows indicate the percentage of increase with respect to the reference group. Abbreviations: control (C), sitosterol (S) at both doses (1 and 3), and cocrystal B (CCB) at both doses (1 and 3). The percentage of increase compared to their reference S group is indicated.

Sitosterol levels determined at endpoint (day 22) are shown in Figure 7. As expected, sitosterol levels increased with phytosterol treatment in all treated animals compared to controls (one-way ANOVA). The increase was greater for those treated with CCB3 compared to CCB1 and S1, and to a lesser extent compared to S3 (one-way ANOVA). Sitosterol levels were 11% higher in B1 animals and 25% higher in B3 animals compared to their references treated with the same dose of commercial sitosterol. No differences were detected between S1 and S3, in contrast to B1 and B3 (one-way ANOVA). Overall, these results are consistent with a trend towards greater sitosterol absorption with the cocrystal formulations, although the magnitude of the effect is moderate.

**Figure 8:**
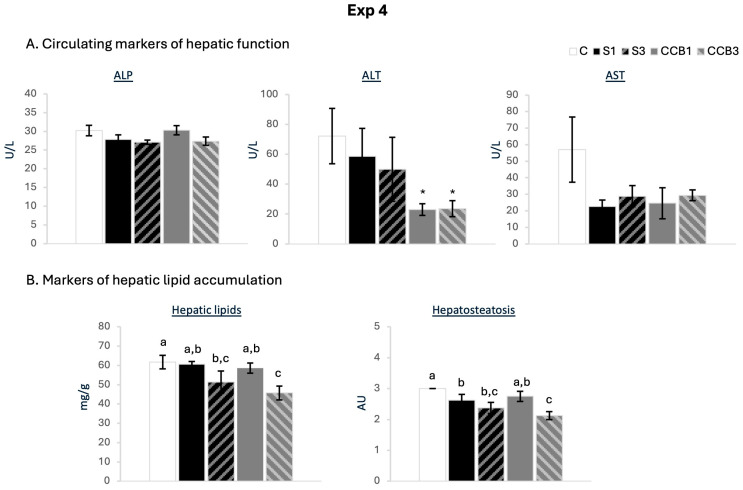
Plasma levels of alkaline phosphatase (ALP), alanine aminotransferase (ALT), and aspartate aminotransferase (AST), as well as the quantitative (mg lipid/g liver) and qualitative assessment of lipids in the liver at the end of the study (day 22) in hamsters from Experiment 4. Data are means ± s.e.m. (n = 8–12). Statistics: one-way ANOVA followed by LSD *post hoc* test was performed (data not sharing a common letter are significantly different); Single comparisons between different groups were performed by Student *t* test: *, different from controls. Abbreviations: control (C), sitosterol (S) at both doses (1 and 3), and cocrystal B (CCB) at both doses (1 and 3).

Figure 8 shows circulating levels of alkaline phosphatase (ALP), alanine aminotransferase (ALT), and aspartate aminotransferase (AST), as well as the quantitative (mg/g) and qualitative assessment of lipids in the liver in the five animal groups after 22 days of treatment (Exp 4). Animals treated with CCB, but not those treated with S, showed a reduction in plasma levels of alanine aminotransferase (ALT) with both doses compared to their controls (Student’s *t*-test). Regarding hepatic lipid content, both S and CCB treatments led to a significant reduction for high doses and to a partial reduction for low doses in liver lipids (mg/g) compared to the controls (one-way ANOVA). Morphological analysis showed similar results, although the hepatic steatosis-reducing effect was observed in S1, S3, and B3 groups compared to controls (one-way ANOVA). This reduction was especially marked in the CCB3 animals, which showed a greater reduction in liver fat. Specifically, with the high dose, hepatosteatosis caused by the consumption of an obesogenic diet was 29% lower in the B3 animals than in the control group, and 11% lower than in the group treated with commercial sitosterol at the same dose.

In Supplementary Figure S2, adipocyte diameter distributions differed significantly among the five experimental groups (χ^2^ test). Pairwise comparisons showed that B1 presented a higher proportion of smaller adipocytes compared with S1 (χ^2^ test), whereas no significant differences were detected between S3 and B3. The analysis of mean adipocyte diameter revealed that S1 had a significantly larger mean diameter than S3 and B3 (one-way ANOVA).

## 4. Discussion

In this study, we show that cocrystallization of β-sitosterol with propionic acid modifies its metabolic effects in vivo compared with the effect of the commercial β-sitosterol formulation. Using a hamster model and combining acute and long-term dietary interventions, we observed that β-sitosterol cocrystals improved postprandial lipid responses, increased circulating sitosterol levels, and reduced hepatic lipid accumulation under specific experimental conditions. Importantly, these effects were not uniform across all settings but depended on the type of cocrystal, the administered dose, and the treatment duration. Together, our results indicate that β-sitosterol with propionic acid cocrystals did not simply exhibit the classical cholesterol-lowering action of β-sitosterol, but can also enhance its metabolic benefits, potentially through changes in intestinal availability, with outcomes that depend on the specific cocrystal formulation and experimental context.

The acute studies indicate that β-sitosterol cocrystals can modulate the postprandial lipid response following a fat load challenge, a relevant metabolic context for cardiovascular risk [3]. In particular, CCA consistently attenuated the triglyceride increase induced by an oral fat load in adult (but not in young) animals, indicating a greater resistance to postprandial hypertriglyceridemia compared with both the control and the commercial β-sitosterol. This is relevant given that postprandial triglyceride excursions, which largely reflect intestinal lipid absorption and chylomicron metabolism, are key contributors to atherogenic dyslipidemia and predict cardiometabolic risk independently of fasting lipids [27,28].

In parallel, CCB led to a consistently smaller increase in postprandial cholesterol levels across the three acute experiments than controls or the commercial reference, suggesting a more pronounced effect on cholesterol handling during the postprandial phase. This aligns with the known capacity of phytosterols to hinder cholesterol incorporation into mixed micelles [10], but the effect surpassed that of the commercial reference at the same dose.

The crystal architecture of CCA and CCB is governed by the complementary contribution of hydrophilic hydrogen-bonding networks and hydrophobic van der Waals interactions, generating a bilayer-like organization with amphiphilic structural features, which could hypothetically influence the dissolution behavior [21]. Because the efficacy of phytosterols is typically limited by their poor solubility and low intestinal availability [11,12], the improved postprandial lipid responses observed with the cocrystals are consistent with differences in intestinal availability of β-sitosterol, although solubility, dissolution behavior, and absorption were not directly assessed in the present study.

Acute treatment with β-sitosterol cocrystals, both A and B, was also associated with a significant reduction in postprandial glucose, but only in the young animals. These findings might reflect a better glucose management capacity of young hamsters treated with the cocrystals, possibly as an indirect consequence of their improved lipid handling. In adolescents and young human adults, a reduction in circulating glucose levels after a fat load challenge is considered a normal physiological response [29,30]. Moreover, in young healthy men but with a paternal history of myocardial infarction, an OFTT detects altered insulin sensitivity, by a reduced decrease in glycemia, despite normal responses in an OGTT [29], supporting the utility of the glycemic response to an OFTT in assessing glucose homeostasis in young individuals. Nonetheless, these findings in our model should be interpreted cautiously, as no glucose-lowering effects were detected in adult hamsters or in the long-term studies. Further research is needed to clarify whether β-sitosterol cocrystals exert a primary influence on glucose metabolism, especially considering evidence that co-administration with metformin may attenuate the latter’s insulin-sensitizing action in obese rats [31].

Notably, the differential acute effects between the two forms of β-sitosterol:propionic acid cocrystals were observed despite identical dosing and testing within the same experimental procedure, supporting the idea that the crystalline form of β-sitosterol influences its biological activity [19,32].

In the long-term intervention under HF diet conditions (Exp 3), CCA induced measurable changes in lipid metabolism over time. Both commercial β-sitosterol and CCA reduced circulating cholesterol levels at the intermediate time point (day 14), despite consuming a diet very rich in fat and cholesterol. Notably, CCA, but not the commercial reference, significantly reduced circulating triglyceride levels at day 14, consistent with the TG-lowering effect observed in acute experiments. These effects, however, were transient and no longer evident at the end of the intervention. This pattern may be related to metabolic adaptations induced by prolonged exposure to obesogenic diets or sitosterol treatment. In rodents fed diets rich in saturated fat and cholesterol, circulating cholesterol levels often reach a plateau within the first weeks and stabilize thereafter [33,34]. Supporting this, previous work showed that male hamsters on HF diets reach a plateau in circulating cholesterol after four weeks, and no longer rise even after prolonged obesogenic-diet feeding [33]. Accordingly, animals in the present study may have reached their cholesterol plateau early in the intervention, limiting the magnitude of further changes and reducing the apparent impact of treatment, as seen in the control animals. Additionally, prolonged phytosterol supplementation can trigger compensatory responses, including increased endogenous cholesterol synthesis and enhanced fecal sterol excretion, which may attenuate the magnitude of cholesterol-lowering effects over time [35].

Despite these adaptations, some indicators suggest improved handling in the treated groups. At the end of the study, body fat content did not increase significantly relative to baseline in CCA- and S- treated animals, whereas control animals showed a significant body fat gain, suggesting a modest but relevant improvement in coping with the lipid overload. Notably, the decrease in cholesterol at the endpoint reached statistical significance only in the CCA group when compared with its own day-14 levels, also in agreement with an improved lipid management by the cocrystal form.

These results suggest that commercial β-sitosterol exerts a stronger initial cholesterol-lowering effect, which diminishes over time under continued obesogenic feeding. In contrast, CCA produces a milder reduction at day 14 but maintains a more persistent effect at the end of the intervention. This pattern, together with the lower relative liver weight observed in CCA-treated animals, supports the idea that CCA may help delay or attenuate diet-induced disturbances in cholesterol homeostasis during chronic HF feeding. Tang et al. investigated a bile acid-based phytosterol cocrystal (PS–CA) for four weeks at two doses in rats under a high-fat diet. The low dose used in that study is similar to the one used in Exp 3 for CCA. The PS–CA showed enhanced efficacy compared to free phytosterols, with greater improvements in lipidemic profile, body weight gain, fat accumulation, and hepatic steatosis. These effects were dose-dependent and attributed to improved bioavailability. In comparison, CCA showed more modest metabolic effects, suggesting that both treatment duration and coformer selection may critically influence in vivo efficacy.

Together, our results of Exp 3 indicate that β-sitosterol cocrystal A may exert mild but biologically relevant effects on lipid management under chronic very HF diet feeding. Further studies are required to determine whether these improvements translate into sustained enhancements in metabolic health.

The responses observed in animals fed a WD in Exp 4 highlight the importance of diet composition and the exact form of cocrystal supplementation in affecting the metabolic effects of phytosterol-based interventions. WDs, characterized by a combination of HF and high sugar content, are known to promote dyslipidemia, hepatic lipid accumulation, and increased body weight through mechanisms that differ from those induced by very HF, low-carbohydrate diets [36,37,38]. Under these conditions, cocrystal B showed persistent cholesterol-lowering effects and improvements in several metabolic parameters, accompanied by a nonsignificant tendency to improve LDL circulating levels. In addition to attenuating cholesterol increases, treatment with CCB was associated with a smaller increase in body weight gain and improvements in hepatic function and adipocyte morphology, suggesting that this formulation may be particularly effective in modulating dietary lipid handling.

The reduced body-weight gain observed with CCB is consistent with other studies indicating that partial attenuation of cholesterol absorption and postprandial lipid handling can indirectly influence energy balance and adiposity during obesogenic feeding [36,38]. In fact, CCB also exerted clear hepatic effects: the higher dose markedly reduced hepatic lipid content, both quantitatively and histologically, and more effectively than the equivalent dose of commercial β-sitosterol. In parallel, animals treated with CCB, but not with commercial β-sitosterol, showed lower plasma ALT levels, suggesting improved liver metabolic status. Hepatic lipid accumulation under obesogenic diets is closely linked to increased intestinal lipid flux and impaired hepatic lipid handling [39]. Interventions that reduce intestinal lipid absorption or modify postprandial lipid delivery to the liver have been shown to attenuate diet-induced steatosis in rodent models [40]. Although the present data do not allow direct conclusions regarding hepatic mechanisms, our findings indicate that cocrystallization of β-sitosterol with propionic acid may extend its metabolic effects beyond circulating cholesterol to include liver lipid homeostasis. Moreover, treatment with CCB was associated with improvements in adipocyte morphology, including reduced adipocyte hypertrophy at the low dose compared with animals treated with the same dose of commercial β-sitosterol. These changes suggest a more favorable pattern of adipose tissue expansion [41], consistent with improvements in liver function.

Overall, for cocrystal B, we found two separate dose-dependent responses. The low dose exerted significant improvements in cholesterol management, diet-induced weight gain, and adipose tissue morphology, whereas the high dose primarily improved liver function [42].

A key finding of the present study is the higher circulating sitosterol levels observed in animals treated with β-sitosterol cocrystals compared with those receiving the commercial reference in both long-term interventions. In Exp 3, CCA significantly increased plasma sitosterol levels relative to both control and commercial β-sitosterol groups, whereas in Exp 4, CCB induced a clear dose-dependent increase in circulating sitosterol, although differences versus equivalent doses of the commercial compound were modest. These results provide in vivo evidence that cocrystallization enhances the systemic availability of β-sitosterol. The low intestinal absorption of phytosterols is well documented and is partly attributed to their poor aqueous solubility and limited incorporation into mixed micelles during digestion [11,42]. Therefore, the higher sitosterolemia observed with the cocrystals indirectly suggests improved intestinal accessibility, which may facilitate a more effective competition with dietary cholesterol at the luminal level. Similar improvements in solubility, dissolution rate, and bioavailability have been reported for other cocrystals [18], including phytosterols with bile acid–based formulations, which were associated with enhanced lipid-lowering efficacy in vivo in rats [23]. However, the present findings should be interpreted with caution as, compared with humans, hamsters appear to show relatively higher systemic exposure to dietary phytosterols, likely due to differences in intestinal transport and biliary excretion mechanisms via ABCG5/G8 transporters [43]. Thus, alterations in circulating sitosterol do not necessarily translate into proportional metabolic efficacy. Indeed, previous work in hamsters has demonstrated that hypocholesterolemic effects of plant sterol analogs can occur independently of ABCG5/G8 expression [44]. Together, these findings indicate that increased sitosterolemia likely contributes as a key mechanism underlying the differential metabolic effects of β-sitosterol cocrystals and support the use of crystal engineering as a strategy to overcome the intrinsic limitations of phytosterols.

Another important observation is that the two cocrystals did not elicit identical metabolic effects. Cocrystal A had a more pronounced impact on postprandial triglyceride handling, whereas cocrystal B consistently improved cholesterol metabolism. These divergent outcomes, obtained under comparable dosing and experimental conditions, support the notion that the specific crystalline form strongly influences biological efficacy. Differences in crystal packing, stability, or dissolution behavior could potentially contribute to these distinct metabolic profiles of CCA and CCB, in line with previous evidence showing that small structural variations among cocrystals can markedly alter solubility and bioavailability [13,21,32,45]. However, the present study did not directly assess these properties in vivo; therefore, no mechanistic conclusions can be drawn. The results also indicate that the classical dose-response behavior described for phytosterols [46] was not clearly reproduced with the cocrystals, in contrast with other findings [23]. While sitosterol absorption increased in a dose-dependent manner, metabolic effects did not fully reflect this pattern, suggesting formulation-dependent behavior beyond simple increases in bioavailability.

Current pharmacological treatments for dyslipidemia, such as statins, fibrates, and ezetimibe, act through mechanisms distinct from phytosterols. While statins and fibrates mainly target hepatic cholesterol synthesis and triglyceride metabolism, respectively [47,48], ezetimibe reduces intestinal cholesterol absorption, inhibiting the NPC1L1 transporter [47]. Phytosterols also act at the intestinal level, but mainly by competing with cholesterol incorporation into mixed micelles [48,49]. Therefore, the effects of β-sitosterol cocrystals should not be directly compared with those of lipid-lowering drugs. Rather, these formulations should be considered as a potential nutraceutical strategy aimed at improving phytosterol performance, particularly in individuals with mild dyslipidemia or as part of dietary interventions.

Some limitations of the present study should be acknowledged. Although circulating sitosterol levels provide indirect evidence of enhanced absorption, future studies should include in vitro dissolution testing, as well as intestinal transport models, to better understand the mechanisms driving the enhanced bioavailability observed. In addition, the strong metabolic adaptations induced by highly obesogenic diets may have attenuated the magnitude of treatment effects, acknowledging the importance of accompanying nutraceutical intervention with improvements in lifestyle habits. Moreover, an additional methodological aspect should be considered in Exp 3. While intermediate samples were taken from peripheral circulation, final samples were obtained via cardiac puncture. Differences between sampling sites may affect lipid concentrations due to dilution and variations in plasma composition [3], which could reduce the power of comparison. Finally, the translational relevance of these findings remains to be established, including their long-term safety, optimal dosing, and the populations most likely to benefit from these cocrystal formulations.

Despite these limitations, the study has several strengths. It combines acute postprandial challenge experiments with long-term dietary interventions and evaluates multiple metabolic outcomes, including circulating lipids, adiposity, hepatic lipid accumulation, and adipose tissue morphology. Moreover, the hamster model used in this study is widely recognized as a valuable model for studying lipoprotein metabolism and cholesterol handling, sharing important physiological similarities with human lipid metabolism [50,51,52], unlike other rodent models.

## 5. Conclusions

To sum up, the present work robustly demonstrates that cocrystallization of β-sitosterol with propionic acid modifies its metabolic effects in vivo, leading to improved postprandial lipid handling, increased systemic availability, and beneficial effects on hepatic health. Importantly, the metabolic responses differed between cocrystal formulations, indicating that not all β-sitosterol cocrystals elicit the same biological effects, even when composed of the same molecular components (or very similar), and underscoring the relevance of the specific crystalline form. These findings support cocrystallization as a promising strategy to overcome the intrinsic limitations of phytosterols and to expand their metabolic potential. Further studies, including translational approaches, will be necessary to establish the relevance of these effects in the human context.

## 6. Patents

R.P., M.P., F.S., and A.P-M. are authors of a patent held by Alimentomica and CIRCE entitled ‘Crystalline Forms Of Beta-Sitosterol’ (PCT/EP2018/066745).

## Figures and Tables

**Table 1:** Experimental design to test the efficacy of two lots of cocrystals.

Experiment	Type	Animal Age	Animals per Group (n)	Dose	Cocrystal Lot	
					CCA	CCB
1.1	Acute	16 w	7	513 mg/kg	144-18	144-19
1.2	Acute	6 w	6	513 mg/kg	144-18	144-19
2	Acute	16 w	7–8	513 mg/kg	192-92	192-47a
3	Chronic	36 w	8	264 mg/kg	192-92	-
4	Chronic	16 w	8–12	114 and 342 mg/kg	-	198-131

Type of study, age of the animals in weeks (w) at the beginning of treatments, number of animals per group in each study, dose used (expressed as mg of β-sitosterol per kg of body weight), and batch of each cocrystal.

**Table 2:** Body weight after 24 h of 40% calorie restriction (T0) and circulating levels of glucose, cholesterol, and triglycerides (TG) at T0 and after 5 h of the fat load (T5) of the acute studies.

		C	S	CCA	CCB	ANOVA
**(** **A) Exp 1.1**						
Body weight (g)	T0	184 ± 9	182 ± 13	183 ± 6	183 ± 11	
Glucose (mg/dL)	T0	88 ± 5	89 ± 4	91 ± 4	83 ± 3	T
	T5	119 ± 10 ^+^	122 ± 7 ^+^	130 ± 6 ^+^	131 ± 9 ^+^	
Cholesterol (mg/dL)	T0	188 ± 18	194 ± 18	174 ± 9	210 ± 15	T
	T5	155 ± 18 ^+^	138 ± 11 ^+^	138 ± 13 ^+^	147 ± 12 ^+^	
Triacylglycerol (mg/dL)	T0	46.5 ± 5.2	47.2 ± 5.2	61.4 ± 7.3	68.2 ± 7.4 *^,#^	T
	T5	99.0 ± 23.7	84.7 ± 18.1 ^+^	57.5 ± 8.5	88.2 ± 8.5	
**(B) Exp 1.2**						
Body weight (g)	T0	119 ± 6	119 ± 4	121 ± 3	122 ± 3	
Glucose (mg/dL)	T0	89 ± 4	91 ± 5	100 ± 5	93 ± 5	T
	T5	83 ± 5	85 ± 5	80 ± 4 ^+^	84 ± 6 ^+^	
Cholesterol (mg/dL)	T0	157 ± 13	136 ± 6	142 ± 14	141 ± 5	T
	T5	116 ± 9 ^+^	133 ± 7	107 ± 6 ^#^	113 ± 4 ^+#^	
Triacylglycerol (mg/dL)	T0	62.7 ± 14.5	79.9 ± 20.2	62.8 ± 22.3	55.3 ± 10.6	T
	T5	176 ± 40 ^+^	121 ± 32	84.4 ± 28.8	105 ± 20	
**(C) Exp 2**						
Body weight (g)	T0	119 ± 6	119 ± 4	121 ± 3	122 ± 3	
Glucose (mg/dL)	T0	99 ± 8	86 ± 5	104 ± 7 ^#*p*=0.060^	97 ± 8	T
	T5	87 ± 4	81 ± 4	90 ± 6	90 ± 7	
Cholesterol (mg/dL)	T0	168 ± 9	160 ± 7	169 ± 15	173 ± 8	
	T5	190 ± 16	178 ± 5 ^+^	162 ± 8	178 ± 13	
Triacylglycerol (mg/dL)	T0	32.7 ± 2.4	46.0 ± 7.1	33.1 ± 4.2	34.9 ± 2.7	TxG
	T5	181 ± 54 ^a+^	103 ± 9 ^a+^	58.2 ± 9.4 ^b+^	98.7 ± 12.2 ^a+^	

Data are means ± s.e.m. (n = 6–8). Statistics: repeated measures ANOVA was performed to analyze the effects of treatment group (G) and/or the time of the measurement (T). At the same time, one-way ANOVA followed by LSD *post hoc* test was performed (data not sharing a common letter are significantly different). Single comparisons between different groups were performed by Student’s *t*-test: *, different from controls; #, different from S group; Single comparisons between T5 and T0 within the same group were performed by Paired *t*-test: +, different from T0. Abbreviations: control (C), sitosterol (S), cocrystal A (CCA), and cocrystal B (CCB).

**Table 3:** Blood glucose and tissue weights obtained at endpoint (D22) of animals from Exp 3.

Exp 3	Glucose (mg/dL)	Liver Weight (%)	rWAT Weight (%)	Heart Weight (%)	Kidney Weight (%)
C	137 ± 17	3.65 ± 0.06	1.14 ± 0.09	0.409 ± 0.020	0.761 ± 0.020
S	124 ± 5	3.51 ± 0.15	0.96 ± 0.05	0.423 ± 0.017	0.772 ± 0.024
CCA	130 ± 11	3.41 ± 0.08 *	1.08 ± 0.06	0.408 ± 0.015	0.755 ± 0.030

Data are means ± s.e.m. (n = 8). Statistics: Single comparisons between different groups were performed by Student’s *t*-test: *, different from controls. Abbreviations: control (C), sitosterol (S), and cocrystal A (CCA).

**Table 4:** Body weight, body fat content, and the levels of glucose, cholesterol, and TG levels at day 1 (14 days after WD and before initiating treatments), day 13, and day 22, as well as the plasma levels of HDL and LDL at the endpoint of hamsters from Exp 4.

Exp 4		C	S1	S3	CCB1	CCB3
Body weight d1	g	136 ± 3	132 ± 3	139 ± 4	140 ± 3	132 ± 2
Body weight d13	g	141 ± 2 ^+^	137 ± 4 ^+^	142 ± 6	142 ± 4	135 ± 3 ^+(*p*=0.061)^
Body weight d22	g	145 ± 3 ^+^^	141 ± 4^+^^	146 ± 5 ^+^^	145 ± 36 ^+^^	139 ± 2 ^+^^
Repeated measures ANOVA		T				
Body fat d1	%	17.2 ± 1.1	18.1 ± 0.7	17.2 ± 0.7	18.6 ± 0.6	17.1 ± 0.9
Body fat d13	%	17.6 ± 1.3	18.3 ± 0.6	17.2 ± 1.7	19.6 ± 1.1	17.9 ± 1.0
Body fat d22	%	17.1 ± 1.4	19.4 ± 1.1	18.6 ± 1.7	19.7 ± 1.4	18.0 ± 1.1
Repeated measures ANOVA		ns				
Glucose d1	mg/dL	102 ± 4	94.6 ± 5.1	92.3 ± 5.0	91.5 ± 5.2	98.3 ± 6.1
Glucose d13	mg/dL	96 ± 4	103 ± 7	106 ± 6	104 ± 7	97 ± 7
Glucose d22	mg/dL	95 ± 9	94 ± 6	85 ± 4	85 ± 4	86 ± 5
Repeated measures ANOVA		T				
Triglycerides d1	mg/dL	194 ± 25	179 ± 29	213 ± 31	213 ± 35	217 ± 40
Triglycerides d13	mg/dL	270 ± 37	253 ± 39	264 ± 51	251 ± 49	215 ± 44
Triglycerides d22	mg/dL	189 ± 19	248 ± 34	239 ± 20^+^	226 ± 34	199 ± 17
Repeated measures ANOVA		ns				
Cholesterol d1	mg/dL	294 ± 9	300 ± 18	287 ± 12	330 ± 9	298 ± 13
Cholesterol d13	mg/dL	333 ± 13 ^a+^	294 ± 7 ^b^	284 ± 12 ^b^	300 ± 6 ^b+^	299 ± 11 ^b^
Cholesterol d22	mg/dL	317 ± 10	315 ± 15	305 ± 21	307 ± 19	313 ± 15
Repeated measures ANOVA		GxT				
HDL d22	mg/dL	103 ± 10	117 ± 16	99.4 ± 15.0	112 ± 12	111 ± 8
LDL d22	mg/dL	222 ± 14	198 ± 22	205 ± 23	177 ± 8 *^(*p*=0.064)^	201 ± 18

Data are means ± s.e.m. (n = 8–12). Statistics: repeated measures ANOVA was performed to analyze the effects of treatment group (G) and/or the three days studied (T). Within the same day, one-way ANOVA followed by LSD *post hoc* test was performed (data not sharing a common letter are significantly different). Single comparisons between different groups were performed by Student *t* test: *, different from controls. Single comparisons between different days of measurement within the same group were performed by Paired *t* test: +, different from D1; ^, different from D13. Abbreviations: control (C), sitosterol (S), cocrystal A (CCA) and cocrystal B (CCB), non-significant (ns).

## Data Availability

The data presented in this study are available on request from the corresponding author due to privacy reasons.

## Supplementary Materials

The following supporting information can be downloaded at: https://www.mdpi.com/article/10.3390/nu18132146/s1, Supplementary Table S1. Composition of standard diet (SD), high-fat diet (HFD), and western diet (WD). Supplementary Table S2. Basal body weight and plasma levels of glucose, cholesterol, and triglyceride of the animals in Exp 4. Supplementary Table S3. Tissue weights relative to the body weight of the animals in Exp 4 were collected at sacrifice (d22). Figure S1: Delta value of % of body weight, body fat gain, and cholesterol plasma levels in hamsters from Experiment 3. Figure S2. Adipocyte diameter distributions and mean adipocyte diameter in rWAT from Experiment 4.
