# Supplementary material for: Cocrystals of β-Sitosterol with Propionic Acid Improve Postprandial Lipid Response and Long-Term Adaptation to Obesogenic Diets in Hamsters, Surpassing the Effects of Commercial β-Sitosterol
Source: Nutrients. 2026 Jul 2;18(13):2146. doi: 10.3390/nu18132146 (PMC13364242; doi:10.3390/nu18132146)
Supplement: Supplementary file 1 [file nutrients-18-02146-s001.zip › nutrients-4364430-supplementary.pdf]

Supplementary material.

Cocrystals of β-Sitosterol with Propionic Acid Improve Postprandial Lipid Response and Long-Term Adaptation to Obesogenic Diets in Hamsters, Surpassing the Effects of
Commercial β-Sitosterol

Mariona Palou[1-5], Bàrbara Reynés[1-5*], Rafel Prohens[6], Fernando Barrera[7], Andreu Palou-March[1-5], Andreu Palou[1-5], Francisca Serra[1-5]

1. Group of Nutrigenomics, Biomarkers and risk Evaluation (NuBE), University of the Balearic Islands, Spain.
2. Institut d'Investigació Sanitària Illes Balears (IdISBa), Spain.
3. CIBER de Fisiopatología de la Obesidad y Nutrición (CIBEROBN), Spain.
4. Artificial Intelligence Research Institute of the Balearic Islands (IAIB), Spain.
5. Alimentómica S.L. (Spin off no. 001 from UIB), 07120, Spain.
6. Laboratory of Organic Chemistry, Faculty of Pharmacy and Food Sciences, University of Barcelona, Avda. Joan XXIII, 08028, Spain.
7. Center for Intelligent Research in Crystal Engineering, S.L. (CIRCE), 07121, Spain.
8. * Correspondence: barbara.reynes@uib.es

Supplementary Table S1. Composition of standard diet (SD), high-fat diet (HFD) and western diet (WD).

**Description**	**Standard diet (SD)**	**High-fat diet (HFD)**	**Western diet (WD)**
**Energy density (kcal/g)**	3.33	5.24	4.68
**Macronutrient composition (%; g/100g)**			
**Carbohydrates**	60.4	25.6	50.0
**Lipids**	3.1	34.9	21.0
**Proteins**	16.1	26.2	19.8
**Others (fiber, micronutrients and water)**	21.4	13.3	9.4
**Caloric information**	**(%; Kcal/100 Kcal)**		
**Carbohydrates**	72.4	20	40.0
**Lipids**	8.4	60	43.0
**Proteins**	19.3	20	17.0

Supplementary Table S2. Basal body weight ans plasma levels of glucose, cholesterol, and triglyceride of the animals in Exp 4 (before the beginning of the WD)

**Exp 4**	Body weight (g)	Glucose (mg/dl)	Cholesterol (mg/dl)	Triglycerides (mg/dl)
C	133± 4	109± 4	208± 10	140± 14
S1	129± 3	116± 4	203± 6	126± 12
S3	132± 3	119± 2	199± 5	137± 18
CCB1	134± 4	117± 3	202± 7	117± 7
CCB3	132± 3	117± 2	194± 10	120± 10

Supplementary Table S3. Tissue weights relative to the body weight of the animals in Exp 4 collected at sacrifice (d22).

**Exp 4**	Liver weight (%)	rWAT weight (%)	Heart weight (%)	Kidney weight (%)
C	5.11±0.17	0.989±0.090	0.428±0.018	0.898±0.061
S1	5.18±0.10	1.11±0.09	0.394±0.013	0.841±0.022
S3	4.72±0.09	1.06±0.11	0.396±0.019	0.847±0.023
CB1	5.17±0.11	1.24±0.12	0.393±0.009	0.841±0.019
CB3	4.91±0.13	1.05±0.05	0.406±0.016	0.806±0.027

Supplementary Figure S1

Exp 3

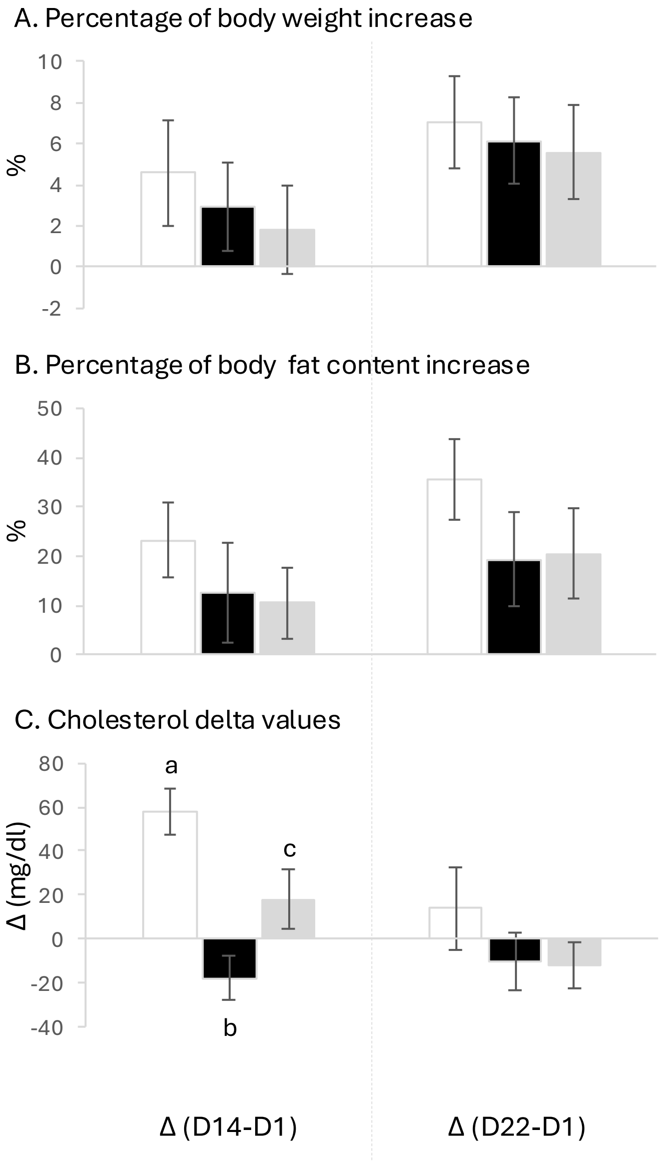

Supplementary Figure S2

Exp 4

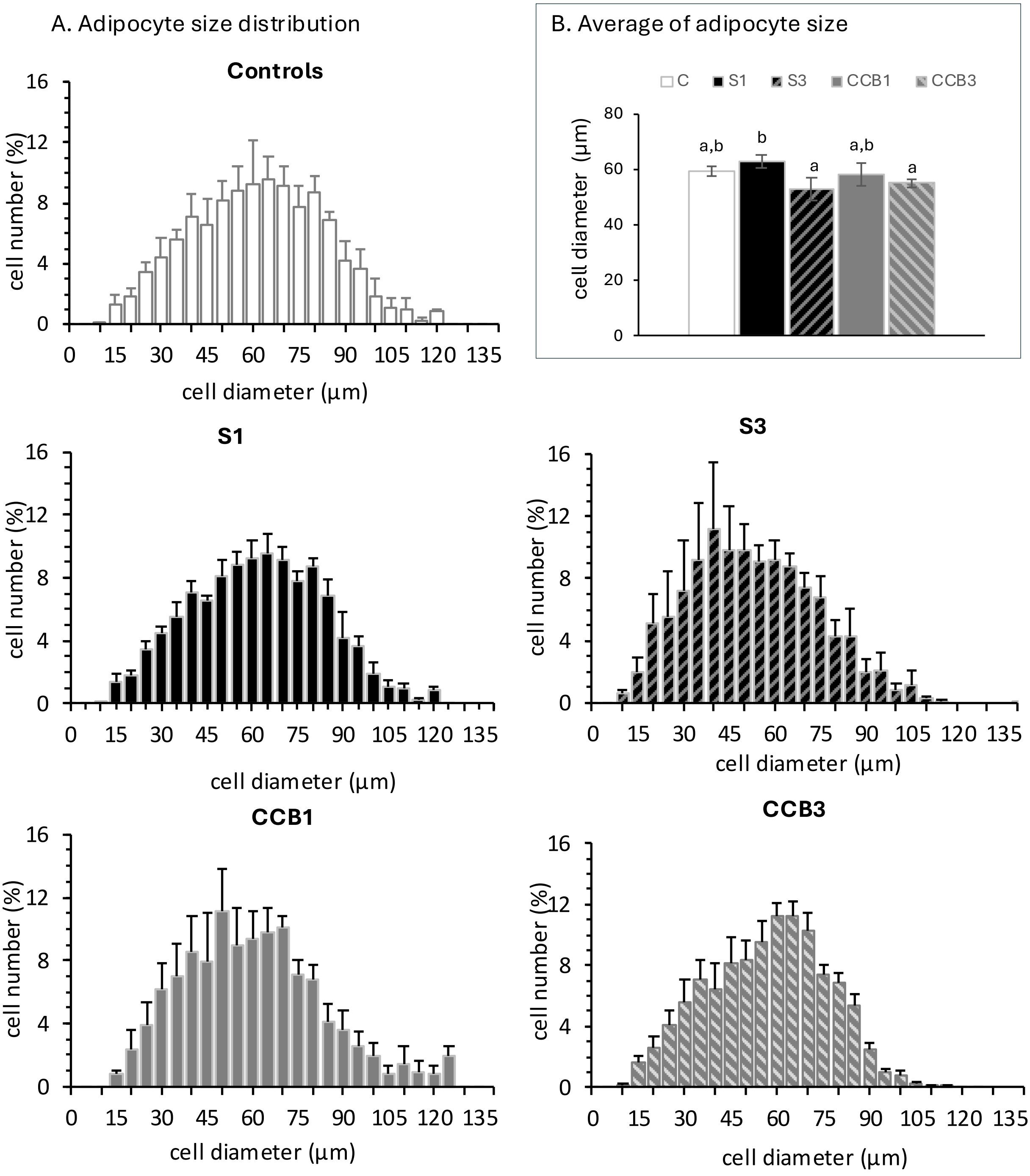

χ^2 B1 *vs* S1 <0.05

χ^2 B3 *vs* S3 n.s.
